# Integration of case-based learning and three-dimensional printing for tetralogy of fallot instruction in clinical medical undergraduates: a randomized controlled trial

**DOI:** 10.1186/s12909-024-05583-z

**Published:** 2024-05-24

**Authors:** Jian Zhao, Xin Gong, Jian Ding, Kepin Xiong, Kangle Zhuang, Rui Huang, Shu Li, Huachun Miao

**Affiliations:** 1 Department of Human Anatomy, Wannan Medical College, No.22 West Wenchang Road, Wuhu, 241002 China; 2 Department of Cardio-Thoracic Surgery, Yijishan Hospital of Wannan Medical College, Wuhu, China; 3 Zhuhai Sailner 3D Technology Co., Ltd., Zhuhai, China; 4 School of Basic Medical Sciences, Wannan Medical College, Wuhu, China

**Keywords:** Medical education, Case-based learning, 3D printing, Tetralogy of fallot, Medical undergraduates

## Abstract

**Background:**

Case-based learning (CBL) methods have gained prominence in medical education, proving especially effective for preclinical training in undergraduate medical education. Tetralogy of Fallot (TOF) is a congenital heart disease characterized by four malformations, presenting a challenge in medical education due to the complexity of its anatomical pathology. Three-dimensional printing (3DP), generating physical replicas from data, offers a valuable tool for illustrating intricate anatomical structures and spatial relationships in the classroom. This study explores the integration of 3DP with CBL teaching for clinical medical undergraduates.

**Methods:**

Sixty senior clinical medical undergraduates were randomly assigned to the CBL group and the CBL-3DP group. Computed tomography imaging data from a typical TOF case were exported, processed, and utilized to create four TOF models with a color 3D printer. The CBL group employed CBL teaching methods, while the CBL-3DP group combined CBL with 3D-printed models. Post-class exams and questionnaires assessed the teaching effectiveness of both groups.

**Results:**

The CBL-3DP group exhibited improved performance in post-class examinations, particularly in pathological anatomy and TOF imaging data analysis (*P* < 0.05). Questionnaire responses from the CBL-3DP group indicated enhanced satisfaction with teaching mode, promotion of diagnostic skills, bolstering of self-assurance in managing TOF cases, and cultivation of critical thinking and clinical reasoning abilities (*P* < 0.05). These findings underscore the potential of 3D printed models to augment the effectiveness of CBL, aiding students in mastering instructional content and bolstering their interest and self-confidence in learning.

**Conclusion:**

The fusion of CBL with 3D printing models is feasible and effective in TOF instruction to clinical medical undergraduates, and worthy of popularization and application in medical education, especially for courses involving intricate anatomical components.

**Supplementary Information:**

The online version contains supplementary material available at 10.1186/s12909-024-05583-z.

## Background

Tetralogy of Fallot (TOF) is the most common cyanotic congenital heart disease(CHD) [1]. Characterized by four structural anomalies: ventricular septal defect (VSD), pulmonary stenosis (PS), right ventricular hypertrophy (RVH), and overriding aorta (OA), TOF is a focal point and challenge in medical education. Understanding anatomical spatial structures is pivotal for learning and mastering TOF [2]. Given the constraints of course duration, medical school educators aim to provide students with a comprehensive and intuitive understanding of the disease within a limited timeframe [3].

The case-based learning (CBL) teaching model incorporates a case-based instructional approach that emphasizes typical clinical cases as a guide in student-centered and teacher-facilitated group discussions [4]. The CBL instructional methods have garnered widespread attention in medical education as they are particularly appropriate for preclinical training in undergraduate medical education [5, 6]. The collection of case data, including medical records and examination results, is essential for case construction [7]. The anatomical and hemodynamic consequences of TOF can be determined using ultrasonography, computed tomography (CT), and magnetic resonance imaging techniques. However, understanding the anatomical structures from imaging data is a slow and challenging psychological reconstruction process for undergraduate medical students [8]. Three-dimensional (3D) visualization is valuable for depicting anatomical structures [9]. 3D printing (3DP), which creates physical replicas based on data, facilitates the demonstration of complex anatomical structures and spatial relationships in the classroom [10].

During the classroom session, 3D-printed models offer a convenient means for hands-on demonstration and communication, similar to facing a patient, enhancing the efficiency and specificity of intra-team communication and discussion [11]. In this study, we printed TOF models based on case imaging data, integrated them into CBL teaching, and assessed the effectiveness of classroom instruction.

## Methods

### Research participants

The study employed a prospective, randomized controlled design which received approval from the institutional ethics committee. Senior undergraduate students majoring in clinical medicine at Wannan Medical College were recruited for participation based on predefined inclusion criteria. The researchers implemented recruitment according to the recruitment criteria by contacting the class leaders of the target classes they had previously taught. Notably, these students were in their third year of medical education, with anticipation of progressing to clinical courses in the fourth year, encompassing Internal Medicine, Surgery, Obstetrics, Gynecology, and Pediatrics. Inclusion criteria for participants encompassed the following: (1) proficient communication and comprehension abilities, (2) consistent attendance without absenteeism or truancy, (3) absence of failing grades in prior examinations, and (4) capability to conscientiously fulfill assigned learning tasks. Exclusion criteria were (1) absence from lectures, (2) failure to complete pre-and post-tests, and (3) inadequate completion of questionnaires. For their participation in the study, Students were provided access to the e-book “Localized Anatomy,” authored by the investigators, as an incentive for their participation. Voluntary and anonymous participation was emphasized, with participants retaining the right to withdraw from the study at any time without providing a reason.

The study was conducted between May 1st, 2023, and June 30, 2023, from recruitment to completion of data collection. Drawing upon insights gained from a previous analogous investigation which yielded an effect size of 0.95 [10]. Sample size was computed, guided by a statistical consultant, with the aim of 0.85 power value, predicated on an effect size of 0.8 and a margin of error set at 0.05. A minimum of 30 participants per group was calculated using G*Power software (latest ver. 3.1.9.7; Heinrich-Heine-Universität Düsseldorf, Düsseldorf, Germany), resulting in the recruitment of a total of 60 undergraduate students. Each participant was assigned an identification number, with codes placed in boxes. Codes drawn from the boxes determined allocation to either the CBL group or the CBL-3DP group. Subsequently, participants were randomly assigned to either the CBL group, receiving instruction utilizing the CBL methodology, or the CBL-3DP group, which received instruction integrating both CBL and 3D Printed models.

### Printing of TOF models

**Fig. 1:**
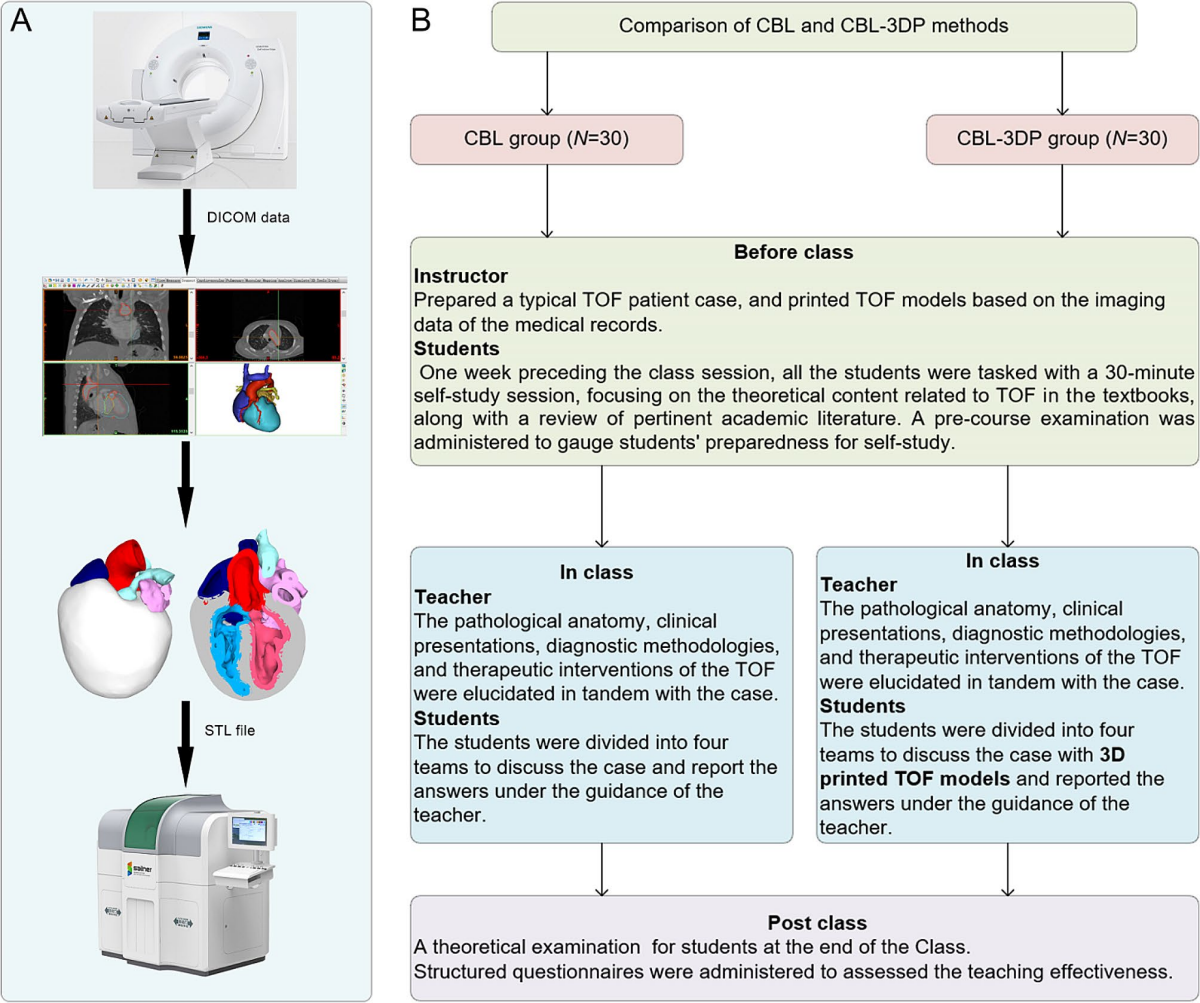
Experimental flow chart of this study. **A** TOF model printing flow chart. **B** The instructional framework

Figure 1A shows the printing flowchart of the TOF models. A typical TOF case was collected from the Yijishan Hospital of Wannan Medical College. The CT angiography imaging data of the case was exported. Mimics Research 20.0 software (Mimics Innovation Suite version 20, Materialize, Belgium) was used for data processing. The cardiovascular module of the CT-Heart tool was employed to adjust the threshold range, independently obtain the cardiac chambers and vessels, post-process the chambers and vessels to generate a hollow blood pool, and merge it with the myocardial volume to construct a complete heart model. The file was imported into Magics 24.0 software (version 24.0; Materialize, Belgium) for correction using the Shell tool page. After repairs, the model entered the smoothing page, where tools such as triangular surface simplification, local smoothing, refinement and smoothing, subdivision of components, and mesh painting were utilized to achieve varying degrees of smoothness. Finally, optimized data were obtained and exported as stereolithography (STL) files. An experienced cardiothoracic surgeon validated the anatomical accuracy of the digital model.

 The STL files were imported into a 3D printer (J401Pro; Sailner 3D Technology, China) for model printing. This printer can produce full-color medical models using different materials. The models were fabricated using two distinct materials: rigid and flexible. Both materials are suitable for the observational discussion of the teaching objectives outlined in our study. From the perspective of observing pathological changes in the TOF, there is no significant difference between the two materials.

### Teaching implementation

Figure 1B illustrates the instructional framework employed in this study. One week preceding the class session, all the students were tasked with a 30-minute self-study session, focusing on the theoretical content related to TOF as outlined in the Pediatrics and Surgery textbooks, along with a review of pertinent academic literature. Both groups received co-supervision from two basic medicine lecturers boasting over a decade of teaching experience, alongside a senior cardiothoracic surgeon. Teaching conditions remained consistent across groups, encompassing uniform assessment criteria and adherence to predefined teaching time frames, all conducted in a Project-Based Learning (PBL) classroom at Wannan Medical College. Additionally, a pre-course examination was administered to gauge students’ preparedness for self-study.

In adherence to the curriculum guidelines, the teaching objectives aimed to empower students to master TOF’s clinical manifestations, diagnostic modalities, and differential diagnoses, while acquainting them with treatment principles and surgical methodologies. Additionally, the objectives sought to cultivate students’ clinical reasoning abilities and problem-solving skills. the duration of instruction for the TOF theory session was standardized to 25 min. The didactic content was integrated with the TOF case study to construct a coherent pedagogical structure.

**Fig. 2:**
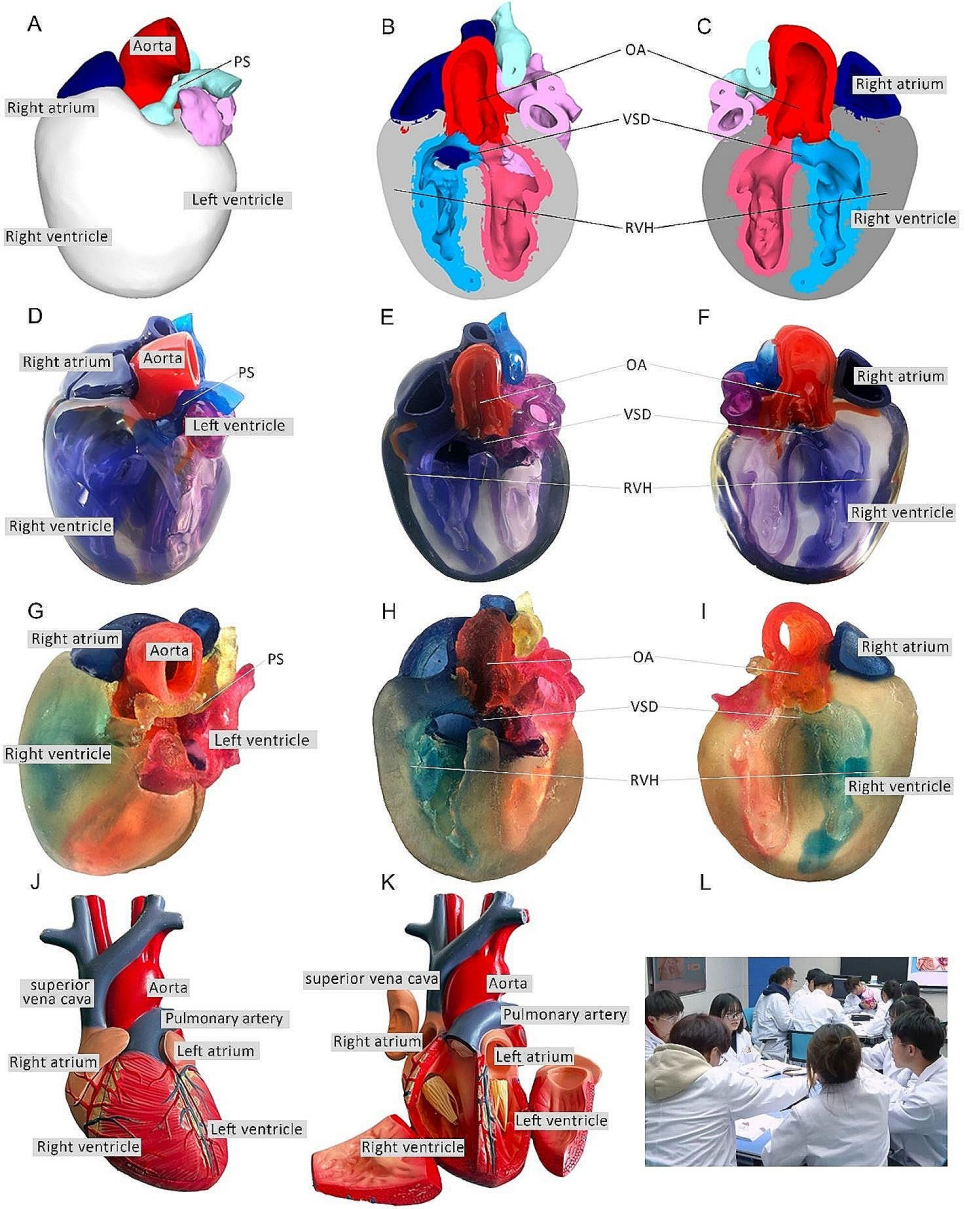
Heart models utilized in instructional sessions. **A** External perspective of 3D digital models. **B, C** Cross-sectional views following trans-septal sagittal dissection of the 3D digital model (PS: Pulmonary Stenosis; OA: Overriding Aorta; VSD: Ventricular Septal Defect; RVH: Right Ventricular Hypertrophy). **D** External depiction of rigid 3D printed model. **E, F** Sagittal sections of the rigid 3D printed model. **G** External portrayal of flexible 3D printed model. **H, I** Sagittal sections of the flexible 3D printed model. **J, K** The normal heart model employed in the instruction of the CBL group. **L** Ongoing classroom session

During the instructional session, both groups underwent teaching utilizing the CBL methodology. Clinical manifestations and case details of TOF cases were presented to stimulate students’ interest and curiosity. Subsequently, the theory of TOF, including its etiology, pathogenesis, pathologic anatomy, clinical manifestations, diagnostic methods, and therapeutic interventions, was briefly elucidated. Emphasis was then placed on the case, wherein selected typical TOF cases were explained, guiding students in analysis and discussion. Students were organized into four teams under the instructors’ supervision, fostering cooperative learning and communication, thereby deepening their understanding of the disease through continuous inquiry and exploration (Fig. 2L). In the routinely equipped PBL classroom with standard heart models (Fig. 2J, K), all students had prior exposure to human anatomy and were familiar with these models. Both groups were provided with four standard heart models for reference, while the CBL-3DP group received additional four 3D-printed models depicting TOF anomalies, enriching their learning experience (Fig. 2D, G). After the lesson, summarization, and feedback sessions were conducted to consolidate group discussions’ outcomes, evaluate teaching effectiveness, and assess learning outcomes.

### Teaching effectiveness assessment

Following the instructional session, participants from the two groups underwent a theoretical examination to assess their comprehension of the taught material. This assessment covered domains such as pathological anatomy, clinical manifestations, imaging data interpretation, diagnosis, and treatment relevant to TOF. Additionally, structured questionnaires were administered to evaluate the efficacy of the pedagogical approach employed. The questionnaire consisted of six questions designed to gauge participants’ understanding of the teaching content, enhancement of diagnostic skills, cultivation of critical thinking and clinical reasoning abilities, bolstering of confidence in managing TOF cases, satisfaction with the teaching mode, and satisfaction with the CBL methodology.

The questionnaire employed a 5-point Likert scale to gauge responses, with 5 indicating “strongly satisfied/agree,” 4 for “satisfied/agree,” 3 denoting “neutral,” 2 reflecting “dissatisfied/disagree,” and 1 indicating “strongly dissatisfied/disagree.” It comprised six questions, with the initial two probing participants’ knowledge acquisition, questions 3 and 4 exploring satisfaction regarding enhanced competence, and the final two assessing satisfaction with teaching methods and modes. Additionally, participants were encouraged to provide suggestions at the end of the questionnaire. To ensure the questionnaire’s validity, five esteemed lecturers in basic medical sciences with more than 10 years of experience verified its content and assessed its Content Validity Ratio and Content Validity Index to ensure alignment with the study’s objectives.

### Statistical analysis

Statistical analyses were conducted utilizing GraphPad Prism 9.0 software. Aggregate score data for both groups were presented as mean ± standard deviation (x ± s). The gender comparisons were analyzed with the chi-square (χ2) test, while the other variables were compared using the Mann-Whitney U test. The threshold for determining statistical significance was set at *P* < 0.05.

## Results

### Three-dimensional printing models

After configuring the structural colors of each component (Fig. 2A, B, C), we printed four color TOF models using both rigid and flexible materials, resulting in four life-sized TOF models. Two color TOF models were created using rigid materials (Fig. 2D, E, F). These models, exhibiting resistance to deformation, and with a firm texture, smooth and glossy surface, and good transparency, allowing visibility of the internal structures, were deemed conducive to teaching and observation. We also fabricated two color TOF models using flexible materials (Fig. 2G, H, I), characterized by soft texture, opacity, and deformability, allowing for easy manipulation and cutting. It has potential utility beyond observational purposes. It can serve as a valuable tool for simulating surgical interventions and may be employed to create tomographic anatomical specimens. In this study, both material models were suitable for observation in the classroom. The participants were able to discern the four pathological changes characteristic of TOF from surface examination or cross-sectional analysis.

### Baseline characteristics of the students

In total, 60 students were included in this study. The CBL group comprised 30 students (14 males and 16 females), with an average age of (21.20 ± 0.76) years. The CBL-3DP group consisted of 30 students (17 males and 13 females) with an average age of 20.96 years. All the students completed the study procedures. There were no significant differences in age, sex ratio, or pre-class exam scores between the two groups (*P* > 0.05), indicating that the baseline scores between the two groups were comparable (Table 1).

**Table 1:** Comparison of the baseline characteristics

	CBL group (*N* = 30)	CBL-3DP group (*N* = 30)	T (χ2) value	*P* value
*Sex*				
Male (n, %)	14 (46.67%)	17 (56.67%)	0.601	0.438
Female (n, %)	16(53.33%)	13 (43.33%)		
Age(years)	21.20 ± 0.76	20.80 ± 0.96	1.787	0.079
Pre-class exam score (20 points)	13.30 ± 1.90	13.50 ± 1.76	0.424	0.673

### Theoretical examination results

All students completed the research procedures as planned. The post-class theoretical examination encompassed assessment of pathological anatomy, clinical presentations, imaging data interpretation, diagnosis, and treatment pertinent to TOF. Notably, no statistically significant disparities were observed in the scores on clinical manifestations, diagnosis and treatment components between the cohorts, as delineated in Table 2. Conversely, discernible distinctions were evident whereby the CBL-3DP group outperformed the CBL group notably in pathological anatomy, imaging data interpretation, and overall aggregate scores (*P* < 0.05).

**Table 2:** Comparison of theoretical exam scores between the two groups

Item	CBL group (*N* = 30)	CBL-3DP group(*N* = 30)	T value	*P* value
Pathological anatomy (10 points)	7.767 ± 1.223	8.933 ± 0.980	4.077	< 0.001
Clinical manifestations (10 points)	8.133 ± 1.383	8.667 ± 1.028	1.695	0.095
Imaging data interpretation (10 points)	7.167 ± 1.289	8.433 ± 1.194	3.948	< 0.001
Diagnosis and treatment (10 points)	7.967 ± 1.351	8.267 ± 1.230	0.899	0.372
Total score (40 points)	31.030 ± 4.590	34.300 ± 3.292	3.168	0.002

### Results of the questionnaires

**Fig. 3:**
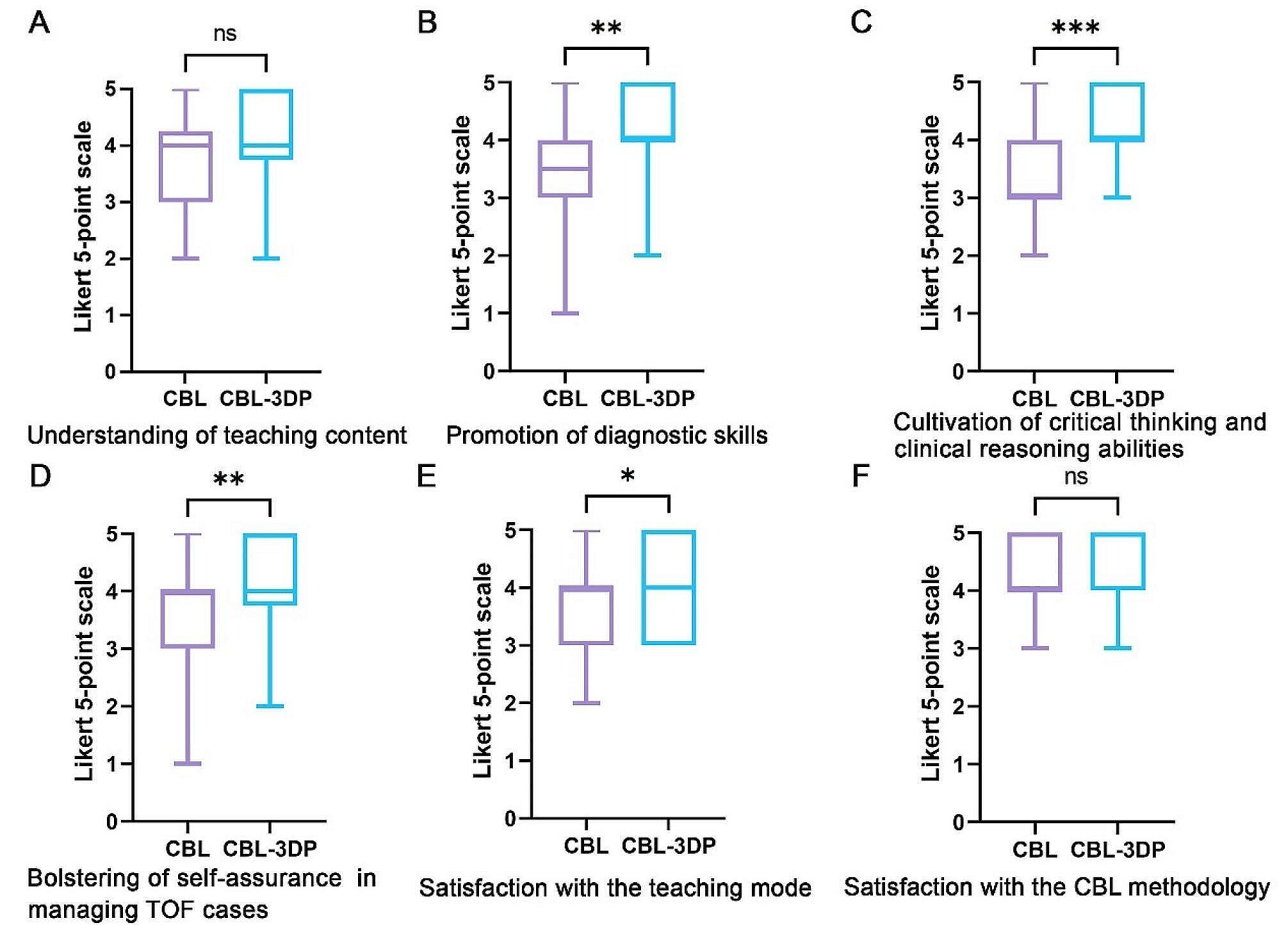
Five-point Likert scores of students’ attitudes in CBL (*n* = 30) and CBL-3DP (*n* = 30) groups. **A** Understanding of teaching content. **B** Promotion of diagnostic skills. **C** Cultivation of critical thinking and clinical reasoning abilities. **D** Bolstering of self-assurance in managing TOF cases. **E** Satisfaction with the teaching mode. **F** Satisfaction with the CBL methodology. *ns* No significant difference, *p < 0.05, **p < 0.01, ***p < 0.001

All the 60 participants submitted the questionnaire. Comparing the CBL and CBL-3DP groups, the scores from the CBL-3DP group showed significant improvements in many areas. This included satisfaction with the teaching mode, promotion of diagnostic skills, bolstering of self-assurance in managing TOF cases, and cultivation of critical thinking and clinical reasoning abilities (Fig. 3B, C, D, E). All of which improved significantly (*P* < 0.05 for the first aspects and *P* < 0.01 for the rest). However, the two groups were not comparable (*P* > 0.05) in terms of understanding of the teaching content and Satisfaction with the CBL methodology (Fig. 3A, F).

Upon completion of the questionnaires, participants were invited to proffer recommendations. Notably, in the CBL group, seven students expressed challenges in comprehending TOF and indicated a need for additional time for consolidation to enhance understanding. Conversely, within the CBL-3DP group, twelve students advocated for the augmentation of model repertoire and the expansion of disease-related data collection to bolster pedagogical efficacy across other didactic domains.

## Discussion

TOF presents a significant challenge in clinical practice, necessitating a comprehensive understanding for effective diagnosis and treatment [12]. Traditional teaching methods in medical schools have relied on conventional resources such as textbooks, 2D illustrations, cadaver dissections, and radiographic materials to impart knowledge about complex conditions like TOF [13]. However, the limitations of these methods in fully engaging students and bridging the gap between theoretical knowledge and practical application have prompted a need for innovative instructional approaches.

CBL has emerged as a valuable tool in medical education, offering students opportunities to engage with authentic clinical cases through group discussions and inquiry-based learning [14]. By actively involving students in problem-solving and decision-making processes, CBL facilitates the application of theoretical knowledge to real-world scenarios, thus better-preparing students for future clinical practice [15]. Our investigation revealed that both groups of students exhibited comparable levels of satisfaction with the CBL methodology, devoid of discernible disparities.

CHD presents a formidable challenge due to the intricate nature of anatomical anomalies, the diverse spectrum of conditions, and individual variations [16]. Utilizing 3D-printed physical models, derived from patient imaging data, can significantly enhance comprehension of complex anatomical structures [17]. These models have proven invaluable in guiding surgical planning, providing training for junior or inexperienced pediatric residents, and educating healthcare professionals and parents of patients [18]. Studies indicate that as much as 50% of pediatric surgical decisions can be influenced by the insights gained from 3D printed models [19]. By providing tangible, anatomically accurate models, 3D printing offers a unique opportunity for people to visualize complex structures and enhance their understanding of anatomical intricacies. Our study utilized full-color, to-scale 3D printed models to illustrate the structural abnormalities associated with TOF, thereby enriching classroom sessions and facilitating a deeper comprehension of the condition.

Comparative analysis between the CBL-3DP group and the CBL group revealed significant improvements in post-test performance, particularly in pathological anatomy and imaging data interpretation. Additionally, questionnaire responses indicated higher levels of satisfaction and confidence among students in the CBL-3DP group, highlighting the positive impact of incorporating 3D printed models into the learning environment, improving the effectiveness of CBL classroom instruction. Despite the merits, our study has limitations. Primarily, participants were exclusively drawn from the same grade level within a single college, possibly engendering bias owing to shared learning backgrounds. Future research could further strengthen these findings by expanding the sample size and including long-term follow-up to assess the retention of knowledge and skills. Additionally, the influence of the 3D models depicting a normal heart on the learning process and its potential to introduce bias into the results warrants consideration, highlighting a need for scrutiny in subsequent studies.

As medical science continues to advance, the need for effective teaching methods becomes increasingly paramount. Our study underscores the potential of combining active learning approaches like CBL with innovative technologies such as 3D printing to enhance teaching effectiveness, improve knowledge acquisition, and foster students’ confidence and enthusiasm in pursuing clinical careers. Moving forward, further research and integration of such methodologies are essential for meeting the evolving demands of medical education, especially in areas involving complex anatomical understanding.

## Conclusions

Integrating 3D-printed models with the CBL method is feasible and effective in TOF instruction. The demonstrated success of this method warrants broad implementation in medical education, particularly for complex anatomical topics.

### Electronic supplementary material

Below is the link to the electronic supplementary material.

Supplementary Material 1

Supplementary Material 2

Supplementary Material 3

## Data Availability

All data supporting the conclusions of this research are available upon reasonable request from the corresponding author.
