# Supplementary material for: Integration of case-based learning and three-dimensional printing for tetralogy of fallot instruction in clinical medical undergraduates: a randomized controlled trial
Source: BMC Med Educ. 2024 May 24;24:571. doi: 10.1186/s12909-024-05583-z (PMC11127445; doi:10.1186/s12909-024-05583-z)
Supplement: Supplementary file 1 — Supplementary Material 1 [file 12909_2024_5583_MOESM1_ESM.docx]

**Date: ID: Age: Sex: Scores:**

**Tetralogy of Fallot Course Pre-Course Test**

**Multiple Choice Directions: Read each question carefully and choose the best answer. Write the letter of your correct answer on the blank. Multiple choice questions worth 2 points each.**

_____1. the primitive heart begins to form in what week of the embryo?

**A. Week 2** B. week 3

C. week 4 D. week 5

_____2. The normal fetal circulation is

A. most of the blood returning from the inferior vena cava to the right atrium flows into the right ventricle.

B. most of the blood from the superior vena cava returning to the right atrium flows into the left atrium

C. most of the blood from the pulmonary artery flows into the lungs

**D. blood from the pulmonary artery may flow into the aorta**

_____3. The normal anatomy of the heart is

A. the aorta is attached to the right ventricle

B. the pulmonary artery is attached to the left ventricle

**C. the right ventricle can be divided into inflow and outflow tracts**

D. the septum is thicker than the muscular part

_____4、The ovoid fossa of the heart is located in the

A. The posterior wall of the left atrium B. the posterior wall of the right atrium

C. the upper left atrial septum **D. lower right atrial septum**

_____5、The right ventricular inflow tract and outflow tract are demarcated by

A. septal rim meatus **B. supraventricular ridge**

C. anterior tricuspid valve D. anterior papillary muscle

_____6、Which description of the interventricular septum is wrong

A. The interventricular septum is thinnest at the fossa ovalis.

B. The fossa ovalis is the most common site of atrial septal defects.

C. The interventricular septum is divided into muscular and membranous parts.

**D. The membranous part of the septum lacks endocardium.**

_____7. The most common type of congenital heart disease is

**A. ventricular septal defect**

B. Atrial septal defect

C. Arterial duct failure

D. Tetralogy of Fallot

_____8. Which of the following is true about fetal blood circulation

**A. Both the right and left ventricles supply blood to the whole body.**

B. All blood from the inferior vena cava flows to the right ventricle.

C. The umbilical artery has the highest oxygen content.

D. Aortic pressure is greater than pulmonary artery pressure.

**Fill in the blanks. Blanks worth 1 point each.**

The four malformations of the tetralogy of Fallot are:
