# Supplementary material for: Integration of case-based learning and three-dimensional printing for tetralogy of fallot instruction in clinical medical undergraduates: a randomized controlled trial
Source: BMC Med Educ. 2024 May 24;24:571. doi: 10.1186/s12909-024-05583-z (PMC11127445; doi:10.1186/s12909-024-05583-z)
Supplement: Supplementary file 2 — Supplementary Material 2 [file 12909_2024_5583_MOESM2_ESM.docx]

**Date: ID: Scores:**

**Tetralogy of Fallot Course Post-Course Test**

**Part I. pathological anatomy**

**Multiple Choice Directions: Read each question carefully and choose the best answer. Write the letter of your correct answer on the blank. Multiple choice questions worth 2 points each.**

_____1. Which of the following malformations is not part of the tetralogy of Fallot

A. Pulmonary stenosis

B. aortic ride

C. ventricular septal defect

**D. Atrial septal defect**

_____2. Pathologic changes in patients with tetralogy of Fallot may present with

**A. Equal peak systolic pressures in the right and left ventricles**

B. higher systolic pressure in the right ventricle than in the left ventricle

C. Congestive heart failure

D left ventricular hypertrophy

_____3. The blood flow changes in tetralogy of Fallot are correctly characterized by

A. generally a left-to-right shunt

**B. generally a right-to-left shunt**

C increased blood flow in the pulmonary circulation

D decreased blood flow in the physical circulation

_____4. The most common cause of right ventricular outflow tract obstruction in tetralogy of Fallot is

A**.** right ventricular hypertrophy

B. pulmonary valve stenosis

**C. funnel stenosis**

D. mitral stenosis

_____5. The main factor that determines the pathophysiology, severity, and prognosis of a patient with tetralogy of Fallot is

**A.right ventricular outflow tract stenosis**

B.right ventricular hypertrophy

C. size of the ventricular septal defect

D.Mitral stenosis

**Part II. Image Data Interpretation**

**Multiple Choice Directions: Read each question carefully and choose the best answer. Write the letter of your correct answer on the blank. Multiple choice questions worth 2 points each.**

_____1. Tetralogy of Fallot may present with imaging manifestations of

A. Left ventricular hypertrophy

B. Enlarged hilar shadows

**C. boot-shaped cardiac shadow**

D. increased pulmonary blood flow

_____2. Patients with severe tetralogy of Fallot may present with

A. insignificant cyanosis

B. severe aortic stenosis

C. large atrial septal defect

**D. markedly decreased pulmonary blood flow on X-ray**

_____3. Tetralogy of Fallot can be visualized by ultrasound.

A. left ventricular hypertrophy.

B. pulmonary artery crossover.

C. widening of the pulmonary arteries.

**D. The right half of the heart is visualized first on acoustic contrast.**

**The figure below shows an echocardiogram of a patient with tetralogy of Fallot, label the four malformations on the figure（4 points, 1 point each）**


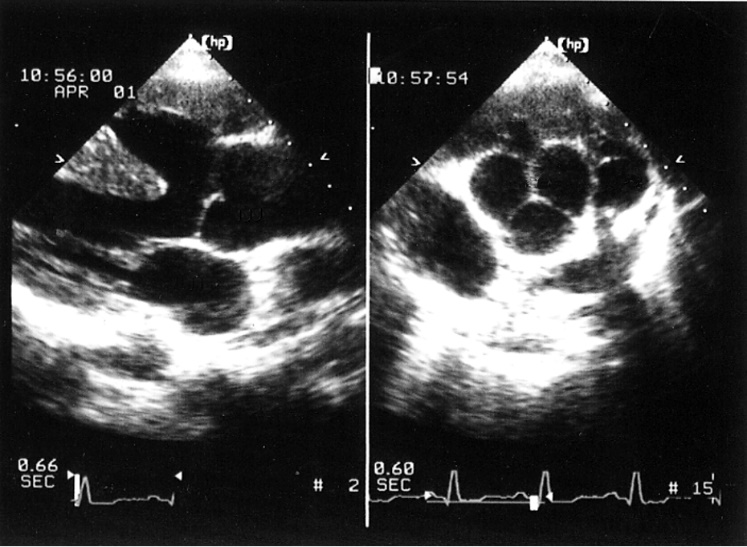


The correct answer:
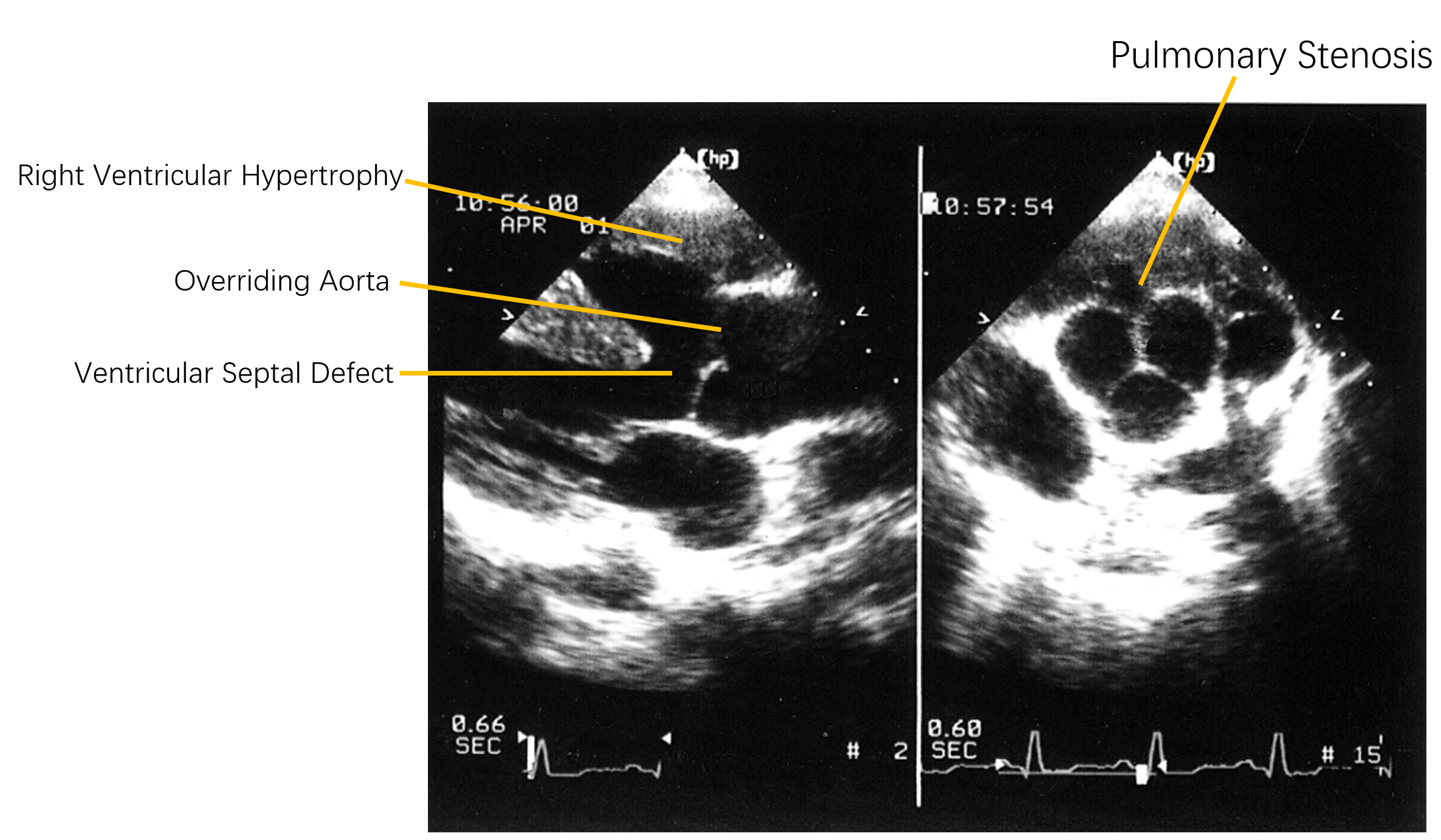


**Part III. Clinical Manifestation**

**Multiple Choice Directions: Read each question carefully and choose the best answer. Write the letter of your correct answer on the blank. Multiple choice questions worth 2 points each.**

_____1. Cyanotic manifestations in patients with tetralogy of Fallot

A. the appearance time is not related to the degree of pulmonary artery stenosis

**B. it is mostly found in the superficial areas with abundant capillaries**

C. the bruising is aggravated when quiet

D. the degree of bruising is related to the degree of aortic stenosis

_____2. Patients with tetralogy of Fallot prefer to squat because of

A. increasing vena cava return

B. increasing pulmonary blood volume and decreasing left-to-right shunting

C. decreasing pulmonary blood volume

**D. increasing resistance in the physical circulation and decreasing right-to-left shunting**

_____3. Which of these is a cause of paroxysmal hypoxia that can occur in patients with tetralogy of Fallot?

**A. pulmonary artery obstruction**

B. aortic obstruction

C. mitral stenosis

D. tricuspid stenosis

_____4. How long does cyanosis persist in a patient with tetralogy of Fallot and how long does it take to develop pestle finger?

A one month

B two months

C half a month

**D six months**

_____5. The most common cause of sudden syncope in children with tetralogy of Fallot is

A. Prolonged hypoxia.

B. Blood clots caused by blood viscosity and slowing down of blood flow.

**C. Muscle spasm in the funnel of the pulmonary artery.**

D. Cerebral abscess.

**Part IV. Diagnosis and Treatment**

**Multiple Choice Directions: Read each question carefully and choose the best answer. Write the letter of your correct answer on the blank. Multiple choice questions worth 2 points each.**

_____1. Indications for surgery in patients with tetralogy of Fallot are

A. left ventricular hypertrophy

**B. well-developed pulmonary arteries**

C. left ventricular end-diastolic volume index <30 ml/m2

D. with severe hepatic and renal impairment

_____2. Surgery in patients with tetralogy of Fallot can

A. reduce pulmonary blood flow

**B. improve arterial oxygen saturation**

C. promote right ventricular development

D. promote aortic development

_____3. It is generally recognized that a systolic right ventricular pressure of more than what is likely to result in myocardial damage.

**A. 50mmHg**

B. 80mmHg

C. 100mmHg

D. 150mmHg

_____4. In asymptomatic or mildly symptomatic patients with tetralogy of Fallot, the current preference is for

A. body-pulmonary circulation shunt

B. valvotomy

C. balloon valvuloplasty

**D. perform elective radical surgery**

_____5. Effective resuscitation measures during hypoxic episodes in tetralogy of Fallot should be except

A. lying position

B. Oxygen administration

**C. Intravenous cediran**

D. Intramuscular injection of phenobarbital sodium
