# Supplementary material for: Integration of case-based learning and three-dimensional printing for tetralogy of fallot instruction in clinical medical undergraduates: a randomized controlled trial
Source: BMC Med Educ. 2024 May 24;24:571. doi: 10.1186/s12909-024-05583-z (PMC11127445; doi:10.1186/s12909-024-05583-z)
Supplement: Supplementary file 3 — Supplementary Material 3 [file 12909_2024_5583_MOESM3_ESM.docx]

**Date: ID: Scores:**

Questionnaire

| **□** | I have received information regarding this research and had an opportunity to ask questions. I believe I understand the purpose, extent, and possible risks of my involvement in this project and I voluntarily consent to take part. |
| --- | --- |

1. The class has improved my understanding of teaching content.

□Strongly agree □Agree □Neutral □Disagree □strongly disagree

2. The class has improved my diagnostic skills.

□Strongly agree □Agree □Neutral □Disagree □strongly disagree

3. The class Cultivated my critical thinking and clinical reasoning abilities

□Strongly agree □Agree □Neutral □Disagree □strongly disagree

4. The class bolstered my self-assurance in managing TOF cases.

□Strongly agree □Agree □Neutral □Disagree □strongly disagree

5. Are you satisfied with the teaching mode?

□Strongly satisfied □Satisfied □Neutral □Dissatisfied □strongly dissatisfied

6. Are you satisfied with the CBL methodology?

□Strongly satisfied □Satisfied □Neutral □Dissatisfied □strongly dissatisfied

7. Would you please give me your valuable advice on this class?
